# Waterpipe smoking induces epigenetic changes in the small airway epithelium

**DOI:** 10.1371/journal.pone.0171112

**Published:** 2017-03-08

**Authors:** Matthew S. Walters, Jacqueline Salit, Jin Hyun Ju, Michelle R. Staudt, Robert J. Kaner, Allison M. Rogalski, Teniola B. Sodeinde, Riyaad Rahim, Yael Strulovici-Barel, Jason G. Mezey, Ahmad M. Almulla, Hisham Sattar, Mai Mahmoud, Ronald G. Crystal

**Affiliations:** 1 Department of Genetic Medicine, Weill Cornell Medical College, New York, New York, United States of America; 2 Department of Medicine, Weill Cornell Medical College, New York, New York, United States of America; 3 Pulmonary Section, Hamad Medical Corporation, Doha, Qatar; 4 Weill Cornell Medical College-Qatar, Doha, Qatar; University of Pittsburgh, UNITED STATES

## Abstract

Waterpipe (also called hookah, shisha, or narghile) smoking is a common form of tobacco use in the Middle East. Its use is becoming more prevalent in Western societies, especially among young adults as an alternative form of tobacco use to traditional cigarettes. While the risk to cigarette smoking is well documented, the risk to waterpipe smoking is not well defined with limited information on its health impact at the epidemiologic, clinical and biologic levels with respect to lung disease. Based on the knowledge that airway epithelial cell DNA methylation is modified in response to cigarette smoke and in cigarette smoking-related lung diseases, we assessed the impact of light-use waterpipe smoking on DNA methylation of the small airway epithelium (SAE) and whether changes in methylation were linked to the transcriptional output of the cells. Small airway epithelium was obtained from 7 nonsmokers and 7 light-use (2.6 ± 1.7 sessions/wk) waterpipe-only smokers. Genome-wide comparison of SAE DNA methylation of waterpipe smokers to nonsmokers identified 727 probesets differentially methylated (fold-change >1.5, p<0.05) representing 673 unique genes. Dominant pathways associated with these epigenetic changes include those linked to G-protein coupled receptor signaling, aryl hydrocarbon receptor signaling and xenobiotic metabolism signaling, all of which have been associated with cigarette smoking and lung disease. Of the genes differentially methylated, 11.3% exhibited a corresponding significant (p<0.05) change in gene expression with enrichment in pathways related to regulation of mRNA translation and protein synthesis (eIF2 signaling and regulation of eIF4 and p70S6K signaling). Overall, these data demonstrate that light-use waterpipe smoking is associated with epigenetic changes and related transcriptional modifications in the SAE, the cell population demonstrating the earliest pathologic abnormalities associated with chronic cigarette smoking.

## Introduction

Waterpipe smoking (also called hookah, shisha, or narghile) is a tobacco use method traditionally associated with the Middle East [1–6]. However, its use is becoming more prevalent in the US and Western societies especially among young adults [4–6]. In contrast to cigarettes, waterpipe smoking involves placing the tobacco in a bowl surrounded by burning charcoal. When the smoker inhales, air is pulled through the charcoal and into the bowl holding the tobacco which results in the smoke being bubbled through water, carried through a hose, and subsequently inhaled [7]. The process of passing the smoke through water leads to a common belief amongst many waterpipe smokers that water filters out “toxins” from the smoke and, therefore, waterpipe is a safer smoking alternative to cigarettes [8]. However, the resulting smoke still includes many volatilized and pyrolyzed tobacco products together with carbon monoxide and charcoal components with the potential to induce toxic effects on the lung [7, 9–16].

We have previously demonstrated that light-use waterpipe smoking by young adults is associated with increased cough and sputum, a reduction in diffusion capacity, increases in blood carboxyhemoglobin, increased levels of pulmonary capillary-derived endothelial microparticles and marked transcriptional changes in alveolar macrophages and the small airway epithelium (SAE) [17]. In the present study, we have assessed the biologic basis of the transcriptional reprogramming induced by waterpipe smoking in the SAE, the initial site of pathologic changes in the lung of cigarette smokers [18–23]. Based on the knowledge that cigarette smoking is associated with modifications in SAE DNA methylation with consequent alterations in the SAE gene expression [24, 25], we asked: is waterpipe smoking also linked to SAE methylation changes, and if so, are there methylation modifications associated with alterations in the SAE transcriptome? Interestingly, the data demonstrates that light-use waterpipe smoking in young adults is associated with a broad range of genome-wide DNA methylation-related changes of the SAE impacting a number of genes linked to pathways previously associated with cigarette smoking. Further, many of these methylation-related changes correlate with waterpipe smoking-associated changes in the SAE transcriptome. Together, these data add to the accumulating evidence that waterpipe smoking is harmful, and may lead to lung disease.

## Methods

### Study population

Self-reported never smokers (“nonsmokers”, n = 7), and self-reported light-use waterpipe-only smokers (“waterpipe smokers”, n = 7) were recruited from the general population in the New York metropolitan area (Table 1). One of the waterpipe smokers had a history of conventional cigarette smoking (less than 5 pack/years) which occurred >20 years prior to enrollment in this study, whereas all other waterpipe smokers and nonsmokers had no history of cigarette smoking. The term “light-use” was used to define waterpipe smokers who reported smoking less than or equal to 5 sessions per week. All subjects were evaluated at the Weill Cornell NIH Clinical and Translational Science Center and Department of Genetic Medicine Clinical Research Facility, using Weill Cornell Institutional Review Board-approved clinical protocols and written informed consent obtained. The criteria for “healthy” was based on medical history, physical exam, complete blood count, coagulation studies, liver function tests, urine studies, chest X-ray, EKG and pulmonary function tests as previously described [17, 24]. All subjects were negative for HIV1 and had normal α1-antitrypsin levels (for full inclusion/exclusion criteria, see S1 Methods). Urine nicotine and cotinine levels were determined using liquid chromatography-tandem mass spectrometry (ARUP laboratories, Salt Lake City, UT) [26].

**Table 1:** Demographics of the study population and biological samples^1^.

Parameter	Nonsmokers	Waterpipe smokers	p value
n	7	7	
Gender (male/female)	3/4	3/4	
Age (years)	30 ± 4	27 ± 9	p>0.42
Race (B/W/O)^2^	3/1/3	3/0/4	
Smoking history			
Age of initiation	-	21 ± 6	
Duration of smoking (yr)	-	5.9 ± 7.8	
Session/wk	-	2.6 ± 1.7	
Urine nicotine (ng/ml)^3^	0	44 ± 50	
Urine cotinine (ng/ml)^3^	0	151 ± 101	
Carboxyhemoglobin (%)	1.3 ± 0.3	2.6 ± 1.5	p>0.06
Pulmonary function parameters^4^			
FVC (% predicted)	104 ± 10	97 ± 12	p>0.2
FEV1 (% predicted)	101 ± 12	99 ± 8	p>0.6
FEV1/FVC (% observed)	81 ± 7	87 ± 6	p>0.1
TLC (% predicted)	90 ± 10	94 ± 9	p>0.4
DLCO (% predicted)	96 ± 13	83 ± 11	p>0.07
Small airway epithelium			
% inflammatory cells	1.4 ± 1.5	0.8 ± 1.1	p>0.4
% epithelial cells^5^	98.6 ± 1.5	99.2 ± 1.1	p>0.4
% ciliated	64.2 ± 7.5	67.6 ± 7.7	p>0.4
% secretory	9.8 ± 5.2	11.7 ± 5.5	p>0.5
% basal	9.0 ± 7.2	3.6 ± 4.0	p>0.1
% intermediate	16.0 ± 4.3	16.2 ± 3.4	p>0.9

^1^ Data presented as average ± standard deviation; p value of numeric parameters calculated by student’s t-test with p < 0.05 being significant.

^2^ Abbreviations: B = Black; W = White; O = Other.

^3^ Undetectable urine nicotine < 2 ng/ml; cotinine < 5 ng/ml.

^4^ Pulmonary function testing parameters are given as % of predicted value with the exception of FEV1/FVC, which is reported as % observed; FVC—forced vital capacity; FEV1—forced expiratory volume in 1 sec; TLC—total lung capacity; DLCO—diffusing capacity. Values are measured pre-bronchodilators.

^5^ As a percentage small airway epithelium recovered (see S1 Table for information on the yield of epithelial cells in each subject).

#### Sampling and processing of the epithelium

Small airway epithelium (SAE; 10^th^ to 12^th^ order) was collected via brushing of the epithelium by fiberoptic bronchoscopy as previously described [17, 20, 21, 24]. Following withdrawal of the bronchoscope, the cells were dislodged from the brush by flicking the brush tip in 5 ml of ice-cold Bronchial Epithelium Basal Medium (BEBM, Lonza, Walkersville, MD) with an aliquot of each sample used to quantify the total number of cells recovered, and to quantify the percentage of epithelial and inflammatory cells and the proportions of epithelial cell subtypes. The remaining SAE cells were then split equally into two separate aliquots and pelleted for subsequent DNA or RNA extraction respectively. DNA was extracted from the SAE (Qiagen Puregene kit, Qiagen, Germantown, MD) and analyzed by spectrophotometry and agarose gel electrophoresis to confirm quality and integrity. Total RNA was extracted from the SAE using the TRIzol method (Invitrogen, Carlsbad, CA) with subsequent clean-up using the RNeasy MinElute RNA purification kit (Qiagen) and stored in RNAsecure (Ambion, Austin, TX) at −80°C. The quality of the RNA was assessed by Bioanalyzer (Agilent Technologies, Santa Clara, CA).

### DNA methylation analysis

To study the effect of waterpipe smoking on the DNA methylation profile of the SAE, the microarray-based high resolution HpaII tiny fragment Enriched by Ligation-mediated PCR (HELP) assay was performed using a 720K Roche-NimbleGen custom array (capturing 117,521 HpaII fragments) on SAE DNA from a total of 14 samples (n = 7 nonsmokers and n = 7 waterpipe smokers) [27]. Quality control of the arrays included assessment of MspI and HpaII intensity distribution and spatial uniformity of the Cy3 and Cy5 signals [28]. For all queried HpaII fragments, intensities were processed to determine the Q centered (Qcent) ratio, and the log_2_ multi-sample, quantile normalized unmethylated / methylated (HpaII/MspI) ratio. The resulting Qcent parameter was exported to Excel with fragment annotation details for statistical analysis. To aid in categorization of the methylation states of specific fragments, the HpaII/MspI ratio was inverted to MspI/HpaII, therefore defining hypomethylated (less methylated) loci with a negative log_2_ ratio value and hypermethylated (increased methylation) loci a positive log_2_ ratio value. The phenotypes were evaluated in Partek Genomics Suite Software version 6.6 (Partek Inc., St. Louis, MO) for sources of variation. A 4-way ANOVA was performed to assess waterpipe smoking on methylation of the 117,521 HpaII fragments, with Qcent ratios corrected by age, gender, ethnicity and region of SAE (left *vs* right lower lobe). Fold-change was determined as: [least square mean waterpipe smokers /least square mean nonsmokers]. Probe fragments with a waterpipe smoking *vs* nonsmoking p value <0.05 calculated by a Student’s t-test and a fold-change >±1.5 were designated as the threshold (no significant probe fragments were identified with a Benjamini Hochberg corrected p value <0.05). The closest gene was determined for these fragments and annotation files were used to map individual HpaII fragments relative to transcription start site of the closest gene. The raw data are publically available at the Gene Expression Omnibus (GEO) site (http://www.ncbi.nlm.nih.gov/geo/), accession number GSE92662.

### Gene expression analysis

To study the effect of waterpipe smoking on the gene expression profile of the SAE assessed for methylation analysis, total RNA from the SAE of the same nonsmokers (n = 7) and waterpipe smokers (n = 7) was processed to generate cDNA and perform genome-wide gene expression analysis using the HG-U133 Plus 2.0 array (Affymetrix, Santa Clara, CA) according to Affymetrix protocols. Overall microarray quality was verified by the criteria: (1) 3'/5' ratio for GAPDH ≤3; and (2) scaling factor ≤10.0 [29]. CEL files were processed by Partek for quality control, identification of outliers, and determination of expression level for all probesets, using Robust Multi-chip Average (RMA) method with Partek default parameters. The phenotypes were evaluated in Partek for sources of variation. A 4-way ANOVA was performed to assess waterpipe smoking on gene expression corrected by age, gender, ethnicity and region of SAE (left *vs* right lower lobe). A p value <0.05 calculated by a Student’s t-test with no fold-change cutoff were designated as the threshold. Validation of the HG-U133 Plus 2.0 array gene expression data by RNA sequencing in a subset of the same samples was carried out as detailed in S1 Methods. The raw data are publically available at the Gene Expression Omnibus (GEO) site (http://www.ncbi.nlm.nih.gov/geo/), accession number GSE92662.

### Correlation of gene expression with methylation

The correlation between waterpipe smoking-dependent DNA methylation and gene expression of the SAE was assessed using a starburst plot comparing the p values for waterpipe smoking-dependent methylation to the p value for smoking-dependent gene expression. This analysis was performed for 673 unique genes from the 727 HpaII probe fragments differentially methylated in waterpipe smokers compared to nonsmokers (p<0.05, fold-change > ±1.5) that also had corresponding HG-U133 Plus 2.0 gene expression data.

### Statistics

Comparison of demographic parameters among groups was performed by two-tailed Student’s t-test. A 4-way ANOVA was performed on the DNA methylation HELP assay data to examine the influence of covariates on waterpipe smoking response. For the HG-U133 Plus 2.0 gene expression data, a 4-way ANOVA was also performed to examine the influence of covariates on smoking response.

## Results

**Fig 1:**
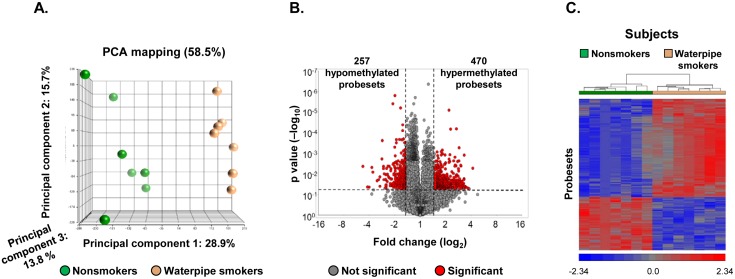
Genome-wide methylation differences of Small Airway Epithelium (SAE) DNA of waterpipe smokers *vs* nonsmokers. The data is derived from analysis of n = 7 nonsmokers and n = 7 waterpipe smokers. **A**. Principal component analysis using all HELP assay probesets corrected for covariates as input dataset. Shown are the first 3 principal components representing the largest variability among the groups. Each circle represents an individual subject (green = nonsmokers, orange = waterpipe smokers). **B**. Volcano plot. Assessment of differential DNA methylation of SAE for all probesets comparing waterpipe smokers *vs* nonsmokers; y-axis, negative log_10_ of p value; x-axis, log_2_-transformed fold-change; red dots are probesets with differential DNA methylation, gray dots are probesets without differential methylation. Differentially methylated probesets with p<0.05, and fold-change of > ±1.5. **C**. Phenotype clustering based on DNA methylation levels. The data was analyzed by Pearson’s dissimilarity hierarchical analysis with an average linkage of waterpipe smokers and nonsmokers based on the DNA methylation of 727 differentially methylated probesets. Probesets having less DNA methylation in waterpipe smokers compared to nonsmokers are represented in blue, more methylation in red and no change in gray. The probesets are represented vertically and the subjects (green = nonsmokers, orange = waterpipe smokers) horizontally.

To study the effect of waterpipe smoking on the DNA methylation profile of the small airway epithelium (SAE), DNA from the SAE of 7 nonsmokers and 7 waterpipe smokers was assessed by the HELP assay (Table 1). Principal component analysis using all methylation probesets as an input dataset demonstrated clear separation of the samples by waterpipe smoking phenotype when corrected by the covariates age, gender, ethnicity and region of SAE (left *vs* right lower lobe, Fig 1A). To identify differentially methylated probesets between waterpipe smokers and nonsmokers we followed the same approach of Pascual et al. [30] and our previous study of cigarette smoking induced DNA methylation of the SAE [24] and considered p<0.05 and a fold-change > ±1.5 as the threshold for analysis. Using this approach, a total of 727 differentially methylated probesets between waterpipe smokers and nonsmokers were identified representing 673 unique genes (see S1 Data File) with approximately 69% of these differentially methylated probesets located within 2 kb of the transcription start site of a gene. Of the 727 significant probesets, 64.6% (470/727) were hypermethylated and 35.4% hypomethylated (257/727; Fig 1B). Unsupervised hierarchical cluster analysis using the 727 waterpipe smoking-dysregulated probesets revealed complete separation of waterpipe smoker and nonsmoker subjects (Fig 1C).

To identify common effects of waterpipe smoking and cigarette smoking on DNA methylation of the SAE, the 727 waterpipe smoking-dysregulated probesets were compared with the 220 cigarette smoking-dysregulated probesets from a previous study from our laboratory focused on cigarette smokers [24]. The results demonstrated an overlap of 20 probesets (representative of 17 unique genes) differentially methylated in the SAE of both waterpipe smokers and cigarette smokers compared to nonsmokers with 19 probesets displaying the same direction of differential methylation in both waterpipe smokers and cigarette smokers compared to nonsmokers (Table 2). For the remaining probeset associated with the gene PARVA (parvin, alpha), hypomethylation was observed in waterpipe smokers and hypermethylation in cigarette smokers (Table 2). A number of genes demonstrating differential methylation in both waterpipe smokers and cigarette smokers included those encoding for enzymes that catalyze reactions involved in drug metabolism, detoxification of electrophilic compounds, and products of oxidative stress, namely CYP1A1 (cytochrome P450, family 1, subfamily A, polypeptide 1), CYP1B1 (cytochrome P450, family 1, subfamily B, polypeptide 1), GSTM1 (glutathione S-transferase Mu 1) and GSTM5 (glutathione S-transferase Mu 5; Table 2) [31, 32]. Overall these data suggest that both waterpipe and cigarette smoking have an overlapping effect on modifying the SAE epigenome.

**Table 2:** Differentially methylated probesets in the Small Airway Epithelium (SAE) of waterpipe smokers and cigarette smokers compared to nonsmokers^1^.

Gene	HELP assay probeset	Waterpipe smokers *vs* nonsmokers		Cigarette smokers *vs* nonsmokers	
		Fold-change^2^	p value	Fold-change^2^	p value
ALS2CL	chr3:46717850–46718164	-2.54	4.1x10^-2^	-1.54	5.1x10^-3^
ARL17A	chr17:44441838–44442698	-1.50	4.0x10^-2^	-1.53	3.9x10^-3^
C11orf34	chr11:112119273–112120535	-1.53	4.7x10^-2^	-1.70	4.4x10^-4^
C1QB	chr1:22980271–22980570	3.32	1.2x10^-3^	1.62	2.1x10^-2^
CFDP1	chr16:75466152–75466937	-4.02	4.3x10^-2^	-2.10	2.0x10^-2^
CYP1A1	chr15:75014046–75014823	-2.05	9.6x10^-3^	-2.25	6.1x10^-7^
CYP1B1	chr2:38304476–38305083	-1.74	1.3x10^-2^	-3.01	7.4x10^-11^
GSTM1	chr1:110229649–110230044	-3.23	2.1x10^-2^	-1.82	1.1x10^-2^
GSTM1	chr1:110231235–110231580	-4.21	4.3x10^-2^	-1.66	4.3x10^-2^
GSTM5	chr1:110254089–110254484	-3.11	1.9x10^-2^	-1.66	1.7x10^-2^
KCNJ15	chr21:39643884–39644330	-1.58	4.4x10^-2^	-1.52	4.4x10^-3^
LOC100134259	chr2:47056280–47057868	2.44	2.4x10^-2^	1.72	1.4x10^-2^
GYG2P1	chrY:14107436–14107803	-1.92	3.4x10^-2^	-2.01	9.3x10^-3^
GYG2P1	chrY:14107083–14107436	-1.60	1.4x10^-2^	-1.87	1.9x10^-2^
PARVA	chr11:12399648–12400632	-1.67	3.1x10^-2^	1.53	7.9x10^-3^
PTPRE	chr10:129704333–129705103	-2.46	2.3x10^-2^	-1.61	2.4x10^-2^
RASGRP4	chr19:38916237–38916680	1.80	1.3x10^-2^	1.79	3.8x10^-2^
RBFOX3	chr17:77480963–77481240	-1.92	3.0x10^-4^	-1.65	1.7x10^-2^
RBFOX3	chr17:77480388–77480963	-2.72	4.3x10^-3^	-1.93	4.6x10^-2^
RIPPLY1	chrX:106148518–106149275	1.58	1.7x10^-2^	1.54	2.1x10^-3^

^1^ Twenty probesets (representative of 17 unique genes) differentially methylated in the SAE of both waterpipe smokers and cigarette smokers compared to nonsmokers [24]. Probesets are listed based on alphabetical order of the associated gene name.

^2^ Fold-change in HELP assay. Negative fold-change represents hypomethylation and positive fold-change represents hypermethylation.

The molecular pathways associated with the 673 genes impacted at the DNA methylation level by waterpipe smoking in the SAE was examined using Ingenuity Pathway Analysis. The analyses demonstrated that within the top 10 canonical pathways impacted by differentially methylated genes in the SAE of waterpipe smokers *vs* nonsmokers, there was significant enrichment of pathways previously associated with cigarette smoking and chronic obstructive pulmonary disease (COPD) including aryl hydrocarbon receptor signaling (15 genes), G-protein coupled receptor signaling (20 genes) and xenobiotic metabolism signaling (20 genes) further suggesting that both waterpipe and cigarette smoking have overlapping effects on the SAE epigenome (Table 3) [24, 25]. In addition to the cigarette smoking associated pathways, there was also enrichment of pathways previously not associated with cigarette smoking and lung disease including cholecystokinin/gastrin-mediated signaling (12 genes, Table 3), suggesting waterpipe smoking impacts the SAE in a unique way relative to traditional cigarette smoking at the DNA methylation level.

**Table 3:** Top 10 canonical pathways impacted by differentially methylated genes in the Small Airway Epithelium (SAE) of waterpipe smokers *vs* nonsmokers^1^.

Pathway	Ratio^2^	Gene names^3^	p value
Role of NFAT in regulation of the immune response	18/171	CSNK1E, JUN, FCGR1B, SOS2, PLCB2, HLA-DQA1, HLA-DQB1, PIK3R5, GNAL, FCGR2A, RELA, FOS, ITPR3, LCK, HLA-DRB5, PLCB3, CD4, GNA15	4.9x10^-6^
Aryl hydrocarbon receptor signaling	15/140	JUN, SRC, GSTT2/GSTT2B, TFF1, ALDH7A1, CYP1A1, ALDH6A1, RELA, GSTT1, FOS, GSTM1, GSTM5, GSTM4, RARG, CYP1B1	2.5x10^-5^
Cholecystokinin/gastrin-mediated signaling	12/101	FOS, JUN, MAPK14, SOS2, SRC, ITPR3, PLCB2, PLCB3, BCAR1, RHOF, RHOA, IL1F10	5.7x10^-5^
G-protein coupled receptor signaling	20/256	SOS2, SRC, PLCB2, PIK3R5, RAP1GAP, PRKAG2, PDE2A, OPRL1, GNAL, MC1R, RELA, CHRM1, GRM4, PLCB3, PDE6B, AVPR1A, ADCY9, GRM6, HRH2, GNA15	1.2x10^-4^
Neuropathic pain signaling in dorsal horn neurons	11/100	FOS, SRC, ITPR3, GRM4, PLCB2, GRIA1, PIK3R5, PLCB3, PRKAG2, PLCL2, GRM6	2.3x10^-4^
CXCR4 signaling	14/152	JUN, SRC, PLCB2, PIK3R5, GNAL, RHOA, FOS, ITPR3, BCAR1, PLCB3, CD4, RHOF, ADCY9, GNA15	2.3x10^-4^
Xenobiotic metabolism signaling	20/271	GAL3ST2, GCLC, MAPK14, GSTT2/GSTT2B, NDST3, ALDH7A1, CYP1A1, ALDH6A1, PIK3R5, ESD, UST, RELA, GSTT1, GSTM1, PPP2R5A, GSTM5, GSTM4, CYP1B1, ABCC3, HS3ST4	2.5x10^-4^
Renin-angiotensin signaling	11/109	FOS, JUN, AGT, MAPK14, SOS2, TPR3, PIK3R5, PRKAG2, ACE, ADCY9, RELA	4.9x10^-4^
GNRH signaling	12/129	FOS,J UN, MAPK14, SOS2, SRC, ITPR3, PLCB2, PLCB3, PRKAG2, ADCY9, RELA, GNA15	5.8x10^-4^
Endothelin-1 signaling	14/172	JUN, MAPK14, SRC, PLCB2, PLA2G4B, PIK3R5, GNAL, FOS, ITPR3, PLCB3, PLCL2, CASP2, ADCY9, GNA15	8.0x10^-4^

^1^ Functional pathway analysis was carried out using Ingenuity Pathway Analysis (http://www.ingenuity.com) on all differentially methylated genes in the SAE of waterpipe smokers *vs* nonsmokers. Pathways are listed based on P values.

^2^ Number of pathway genes differentially methylated in the SAE of waterpipe smokers *vs* nonsmokers compared to the total number of genes in the curated pathway.

^3^ Name of pathway related genes differentially methylated in the SAE of waterpipe smokers *vs* nonsmokers.

**Fig 2:**
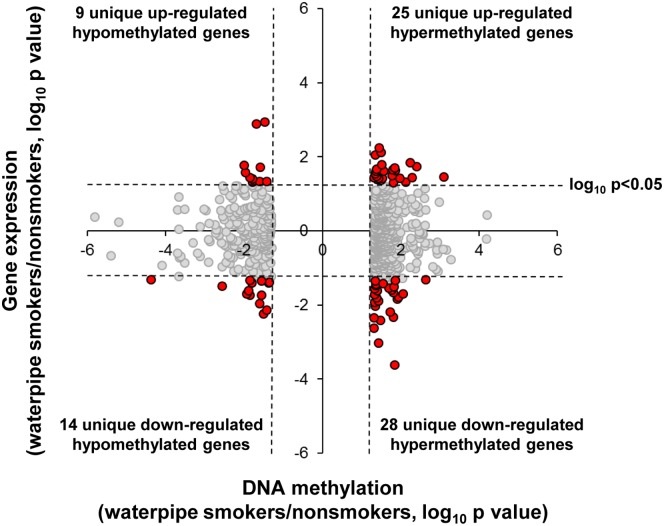
Correlation of Small Airway Epithelium (SAE) DNA methylation and gene expression. Unique genes that show differences in DNA methylation between waterpipe smokers and nonsmokers (p<0.05, fold-change > ±1.5) that also have corresponding gene expression probes on the Affymetrix HG-U133 Plus 2.0 array (673 unique genes from the 727 HpaII probe fragments differentially methylated in waterpipe smokers compared to nonsmokers). The starburst plot shows -log_10_ p value of waterpipe smokers/nonsmokers plotted for DNA methylation (x-axis) *vs* gene expression (y-axis) for each gene. Dashed lines, p values = 0.05. Red dots are probesets with differential DNA methylation and gene expression, gray dots are differentially expressed for DNA methylation but not for gene expression. Left upper quadrant—hypomethylated genes with up-regulated gene expression. Right upper quadrant—hypermethylated genes with up-regulated gene expression. Left lower quadrant—hypomethylated genes with down-regulated gene expression. Right lower quadrant—hypermethylated genes with down-regulated gene expression.

To assess the relationship between waterpipe smoking-dependent DNA methylation and waterpipe smoking-dependent changes in gene expression, a starburst plot was generated examining the 727 differentially methylated probesets (p<0.05, fold-change >±1.5), representing 673 unique genes that had corresponding HG-U133 Plus 2.0 gene expression data (Fig 2). The analysis demonstrated 11.3% (76/673) of the differentially methylated genes displayed a significant change in gene expression (p<0.05, Tables 4 and 5). Validation of these gene expression changes were carried out in a subset of the same samples (n = 3 nonsmokers and n = 3 waterpipe smokers) by RNA sequencing, with comparison of the expression trends between the HG-U133 Plus 2.0 and RNA sequencing gene expression data for 67/76 genes that mapped to unique RefSeq sequences demonstrating a high level of correlation (r^2^ = 0.47, S1 Fig). Of the 76 genes, 23 were hypomethylated, of which 9 were associated with up-regulation of gene expression, and 14 associated with down-regulation (Table 4). For the 53/76 waterpipe smoking-dependent hypermethylated genes, 25 were associated with up-regulation of gene expression and 28 associated with down-regulation (Table 5). Ingenuity Pathway Analysis was performed using the 76 genes demonstrating both waterpipe dependent DNA methylation and gene expression changes as an input dataset to identify impacted molecular pathways. The top two significant canonical pathways impacted at both the DNA methylation and transcriptome level by waterpipe smoking were eIF2 signaling (6 genes) and regulation of eIF4 and p70S6K signaling (4 genes, Table 6) pathways which play an important role in regulating cellular levels of mRNA translation and protein synthesis during homeostasis and in response to environmental stress [33–36].

**Table 4:** Hypomethylated genes in the Small Airway Epithelium (SAE) of waterpipe smokers *vs* nonsmokers with corresponding changes in gene expression^1^.

Gene	HELP assay			U133 gene expression		
	Probeset	Fold-change^2^	p value	Probeset	Fold-change^3^	p value
**Up-regulated gene expression**						
TFF1^4^	chr21:43786117–43786326	-1.51	2.4x10^-2^	205009_at	2.43	4.6x10^-2^
TFF1^4^	chr21:43786326–43786661	-2.26	3.8x10^-2^	205009_at	2.43	4.6x10^-2^
FOLH1	chr11:89396303–89396807	-1.59	1.6x10^-2^	215363_x_at	1.86	3.9x10^-2^
MUC20	chr3:195449743–195450034	-1.63	2.5x10^-2^	1558220_at	1.61	1.9x10^-2^
CHPT1	chr12:102092400–102093324	-1.51	1.6x10^-2^	1559739_at	1.52	4.7x10^-2^
MCTP2	chr15:94942716–94943178	-1.64	2.0x10^-2^	229021_at	1.52	1.3x10^-3^
PRSS16	chr6:27181316–27182057	-1.80	9.9x10^-3^	208165_s_at	1.51	1.6x10^-2^
ADAM28	chr8:24148617–24149755	-1.65	3.3x10^-2^	241446_at	1.42	1.1x10^-3^
SEC16A	chr9:139380018–139380887	-1.51	1.4x10^-2^	215696_s_at	1.25	3.6x10^-2^
RHBDD1	chr2:227700346–227700611	-1.65	1.0x10^-2^	233164_x_at	1.25	2.6x10^-2^
**Down-regulated gene expression**						
CYP1B1	chr2:38304476–38305083	-1.74	1.3x10^-2^	202434_s_at	-1.12	2.4x10^-2^
HOXB8	chr17:46692562–46693181	-1.61	4.1x10^-5^	229667_s_at	-1.12	4.9x10^-2^
SOS2	chr14:50700351–50700856	-1.85	4.1x10^-2^	238830_at	-1.13	4.0x10^-2^
PSMG4	chr6:3260840–3262265	-1.82	2.8x10^-2^	233447_at	-1.17	1.9x10^-2^
HIP1	chr7:75369075–75369603	-1.57	1.4x10^-2^	1560317_s_at	-1.24	1.9x10^-2^
STOML2	chr9:35101706–35102158	-1.61	4.3x10^-2^	215416_s_at	-1.25	4.2x10^-2^
JARID2	chr6:14803688–14803999	-1.51	1.4x10^-2^	203298_s_at	-1.26	4.7x10^-2^
BNIP3	chr10:133798970–133799361	-1.82	1.6x10^-2^	201848_s_at	-1.28	4.1x10^-2^
RPS5	chr19:58894233–58894509	-2.24	3.8x10^-2^	200024_at	-1.29	7.5x10^-3^
QDPR	chr4:17511301–17511919	-1.99	1.1x10^-2^	209123_at	-1.38	2.0x10^-2^
SIP1	chr14:39582245–39583380	-1.71	2.7x10^-3^	211114_x_at	-1.42	3.3x10^-2^
C3orf34	chr3:196433425–196434032	-1.77	2.7x10^-2^	1553158_at	-1.43	4.6x10^-2^
MCL1	chr1:150552326–150554117	-1.55	2.4x10^-2^	214056_at	-1.44	1.1x10^-2^
PARVA	chr11:12399648–12400632	-1.67	3.1x10^-2^	222454_s_at	-1.48	6.0x10^-3^

^1^ Hypomethylated genes in the SAE of waterpipe smokers compared to nonsmokers with corresponding changes in gene expression. Genes are listed based on fold-change of gene expression.

^2^ Fold-change in HELP assay. Negative fold-change represents hypomethylation and positive fold-change represents hypermethylation.

^3^ Fold-change in U133 gene expression. Positive fold-change represents increased gene expression and negative fold-change represents decreased gene expression.

^4^ For TFF1 there are two independent HELP assay probesets that are hypomethylated in the SAE of waterpipe smokers compared to nonsmokers, but a single U133 2.0 microarray probeset.

**Table 5:** Hypermethylated genes in the Small Airway Epithelium (SAE) of waterpipe smokers *vs* nonsmokers with corresponding changes in gene expression^1^.

Gene	HELP assay			U133 gene expression		
	Probeset	Fold-change^2^	p value	Probeset	Fold-change^3^	p value
**Up-regulated gene expression**						
HLA-DQA1	chr6:32604793–32605702	4.19	5.2x10^-3^	213831_at	8.45	3.6x10^-2^
HLA-DQB1	chr6:32631147–32632209	1.77	8.2x10^-4^	209480_at	8.20	3.5x10^-2^
EIF2S1	chr14:67825081–67825321	2.08	1.5x10^-2^	201143_s_at	2.76	1.9x10^-2^
ABCC3	chr17:48711832–48712151	3.04	4.1x10^-3^	208161_s_at	1.57	1.8x10^-2^
CDK10	chr16:89755670–89755932	1.80	1.4x10^-2^	203468_at	1.46	2.3x10^-2^
FAM115A	chr7:143581612–143582257	1.53	3.6x10^-2^	204403_x_at	1.43	5.7x10^-3^
COL16A1	chr1:32170869–32171297	2.04	4.2x10^-2^	204345_at	1.37	3.3x10^-2^
PRKAG2	chr7:151573964–151574355	3.22	3.2x10^-2^	215231_at	1.35	1.6x10^-2^
TMC5	chr16:19474685–19475411	1.56	4.6x10^-2^	240303_at	1.32	2.8x10^-2^
RAP1GAP	chr1:21995005–21995517	2.29	4.2x10^-2^	210618_at	1.32	2.2x10^-2^
CNPY2	chr12:56709384–56709713	1.80	4.6x10^-2^	209797_at	1.30	8.9x10^-3^
ASPHD2	chr22:26824590–26825080	1.62	3.8x10^-2^	227015_at	1.28	3.7x10^-2^
CISD2	chr4:103789518–103790086	1.60	5.9x10^-3^	244275_at	1.26	1.4x10^-2^
SF3B1	chr2:198243286–198243955	1.58	1.7x10^-2^	211185_s_at	1.25	2.2x10^-2^
FAN1	chr15:31109598–31110925	1.62	1.1x10^-2^	239289_x_at	1.23	3.7x10^-2^
FGF1	chr5:141991981–141992881	1.53	1.7x10^-2^	205117_at	1.22	3.1x10^-2^
RARG	chr12:53614211–53614816	2.62	3.0x10^-2^	217178_at	1.21	3.8x10^-2^
STAMBP	chr2:74055331–74056076	1.66	3.2x10^-2^	235361_at	1.20	4.2x10^-2^
EIF2AK4	chr15:40227738–40228469	1.89	1.6x10^-2^	225164_s_at	1.20	4.9x10^-2^
FLJ39739	chr1:147931322–147931613	2.08	4.5x10^-2^	239005_at	1.18	2.4x10^-2^
CDKL2	chr4:76554977–76556043	2.37	7.8x10^-3^	236331_at	1.18	4.8x10^-2^
TTC17	chr11:43380706–43381027	2.00	4.9x10^-2^	224849_at	1.17	3.6x10^-2^
POLR3E	chr16:22308202–22308674	1.76	2.7x10^-2^	233458_at	1.16	2.4x10^-2^
AKAP8	chr19:15490443–15490780	2.58	3.2x10^-2^	203848_at	1.13	7.4x10^-3^
LSM14A	chr19:34663415–34663678	1.85	4.0x10^-2^	222099_s_at	1.12	4.0x10^-2^
**Down-regulated gene expression**						
PTPRS	chr19:5339127–5339748	2.27	2.9x10^-2^	1555666_at	-1.10	3.8x10^-2^
FBXO22	chr15:76196310–76196551	1.97	1.8x10^-2^	225736_at	-1.14	2.6x10^-2^
PRICKLE3	chrX:49043787–49044009	1.54	2.4x10^-3^	217349_s_at	-1.14	4.9x10^-2^
LOC100129716	chr5:90576485–90577374	3.05	4.4x10^-2^	239395_at	-1.14	3.5x10^-2^
ZNF384	chr12:6798919–6799942	1.70	1.2x10^-2^	212369_at	-1.17	1.6x10^-2^
SIRPA	chr20:1876115–1876469	1.81	4.0x10^-2^	202895_s_at	-1.18	2.5x10^-2^
FZD1	chr7:90894525–90894849	2.06	4.2x10^-2^	204452_s_at	-1.19	1.6x10^-2^
SPANXC	chrX:140336315–140336567	1.52	5.0x10^-2^	220217_x_at	-1.21	4.5x10^-3^
WDR33	chr2:128568380–128568877	2.57	4.9x10^-2^	222763_s_at	-1.21	2.4x10^-3^
LOC642597	chr18:5197186–5197418	1.71	4.4x10^-2^	243506_at	-1.22	3.6x10^-2^
RPL26	chr17:8287040–8287521	1.64	1.9x10^-2^	222229_x_at	-1.23	6.6x10^-3^
RAB4B	chr19:41284200–41284788	3.37	4.7x10^-2^	233385_x_at	-1.25	2.8x10^-2^
JARID2	chr6:14738030–14739232	1.50	1.4x10^-2^	203298_s_at	-1.26	4.7x10^-2^
FBXL22	chr15:63893258–63893671	3.29	3.8x10^-2^	241350_at	-1.26	9.5x10^-4^
ZSCAN10	chr16:3142746–3143151	1.95	4.5x10^-2^	1553875_s_at	-1.28	3.7x10^-2^
RPS15	chr19:1438945–1439352	1.81	3.3x10^-2^	200819_s_at	-1.29	3.8x10^-3^
NDUFS8	chr11:67797997–67798357	1.91	4.7x10^-2^	203189_s_at	-1.30	2.0x10^-2^
ATP6V1D	chr14:67825081–67825321	2.08	1.5x10^-2^	208898_at	-1.32	2.4x10^-4^
QDPR	chr4:17511919–17512817	1.66	8.9x10^-3^	209123_at	-1.38	2.0x10^-2^
SNRPB	chr20:2448332–2449291	2.05	1.5x10^-2^	213175_s_at	-1.39	3.2x10^-2^
CBX4	chr17:77815106–77815348	2.39	4.7x10^-2^	206724_at	-1.50	1.2x10^-2^
KGFLP1	chr9:41998251–41999298	1.58	1.6x10^-2^	1554741_s_at	-1.51	2.2x10^-2^
RAB4A	chr1:229406326–229406592	1.54	3.8x10^-2^	203582_s_at	-1.52	1.3x10^-2^
C1orf88	chr1:111888875–111889467	1.84	1.6x10^-2^	228100_at	-1.52	4.7x10^-3^
PA2G4	chr12:56498194–56498668	2.96	2.1x10^-2^	214794_at	-1.52	2.9x10^-2^
UCKL1	chr20:62583662–62583944	2.41	4.5x10^-2^	232675_s_at	-1.54	9.5x10^-3^
UQCC	chr20:34000302–34000646	2.04	1.2x10^-2^	222470_s_at	-1.84	1.5x10^-2^
HBA1	chr16:227144–228315	1.53	4.6x10^-2^	214414_x_at	-8.38	4.6x10^-2^

^1^ Hypermethylated genes in the SAE of waterpipe smokers compared to nonsmokers with corresponding changes in gene expression. Genes are listed based on fold-change of gene expression.

^2^ Fold-change in HELP assay. Negative fold-change represents hypomethylation and positive fold-change represents hypermethylation.

^3^ Fold-change in U133 gene expression. Positive fold-change represents increased gene expression and negative fold-change represents decreased gene expression.

**Table 6:** Top canonical pathways impacted by differentially methylated and expressed genes in the Small Airway Epithelium (SAE) of waterpipe smokers *vs* nonsmokers^1^.

Pathway	Ratio^2^	Gene names^3^	p value
eIF2 signaling	6/76	EIF2S1, SOS2, EIF2AK4, RPS5, RPL26, RPS15	4.3x10^-5^
Regulation of eIF4 and p70S6K signaling	4/76	EIF2S1, SOS2, RPS5, RPS15	1.7x10^-3^

^1^ Functional pathway analysis was carried out using Ingenuity Pathway Analysis (http://www.ingenuity.com) on all differentially methylated and expressed genes in the SAE of waterpipe smokers *vs* nonsmokers. Pathways are listed based on P values.

^2^ Number of pathway genes differentially methylated and expressed in the SAE of waterpipe smokers *vs* nonsmokers compared to the total number of genes in the curated pathway.

^3^ Name of pathway related genes differentially methylated and expressed in the SAE of waterpipe smokers *vs* nonsmokers.

## Discussion

The use of waterpipe to smoke tobacco is increasing worldwide, second only to cigarette smoking [1–6]. Epidemiologic studies in the US, Europe and other countries suggest the increase in prevalence of waterpipe smoking is mainly among young adults and teens, with 10 to 48% of adolescent and young adults admitting to smoking waterpipe, with 10 to 35% being current waterpipe smokers [4–6]. We have previously demonstrated that light-use waterpipe smoking by young adults is associated with a number of abnormal parameters related to lung health including, increased cough and sputum, a reduction in diffusion capacity, increases in blood carboxyhemoglobin, increased levels of pulmonary capillary-derived endothelial microparticles and global changes in the transcriptomes of alveolar macrophages and the small airway epithelium (SAE), two cell populations critical to maintain normal lung health [17]. In the present study, to understand the role of the epigenome in regulating the transcriptomic changes induced by waterpipe smoking in the SAE, we have built on these findings and assessed the effects of waterpipe smoking on the DNA methylation of the SAE, the initial site of pathologic changes in the lung of cigarette smokers [18–23].

### Waterpipe smoking associated methylation changes of the small airway epithelium

DNA methylation, the attachment of methyl groups to cytosine bases followed by guanine (CpG sites), is a heritable and reversible gene regulatory modification that plays a critical role in regulating cell type and tissue-specific gene expression [37, 38]. DNA methylation is highly modified in response to cigarette smoke and altered in multiple lung diseases including asthma, chronic obstructive pulmonary disease (COPD) and idiopathic pulmonary fibrosis (IPF), suggesting that DNA methylation may play an important role in the pathogenesis of these diseases [24, 25, 39–51]. Comparing the SAE DNA methylation of waterpipe smokers to nonsmokers, our results demonstrate that light-use waterpipe smoking is associated with genome-wide DNA methylation changes affecting hundreds of genes. Interestingly, we observed predominant hypermethylation of the affected genes in the SAE of waterpipe smokers relative to the SAE of nonsmokers which contrasts with our previous findings comparing the SAE of cigarette smokers to nonsmokers where predominant hypomethylation was observed [24]. These differences in DNA methylation patterns between waterpipe smoking and cigarette smoking may result from the different chemical composition of waterpipe and cigarette smoke. For example, compared to one cigarette, one waterpipe session exposes the smoker to 2 to 4 times the amount of nicotine, 7 to 11 times the amount of carbon monoxide, 100 times more tar, 17 times the amount of formaldehyde, 2 to 5 times the amount of high molecular weight carcinogenic polyaromatic hydrocarbons and 3 times the amount of phenol [7, 9, 14, 15]. In addition, high levels of benzene, volatile aldehydes and other toxins originating from flavoring have been detected in waterpipe smoke [10–13, 16]. These chemical differences between waterpipe and cigarette smoke likely have differential effects on cellular processes that regulate DNA methylation resulting in differences in global methylation levels and patterns. Despite these global differences, comparison of the effects of waterpipe smoking and cigarette smoking on DNA methylation of the SAE demonstrated an overlap of differentially methylated genes in the SAE of both waterpipe smokers and cigarette smokers compared to nonsmokers suggesting a common effect on modifying the SAE epigenome.

### Alterations in molecular pathways

Characterization of the molecular pathways associated with our waterpipe smoking-dependent differential methylated gene set showed significant enrichment of pathways previously associated with cigarette smoking and COPD in the SAE, including aryl hydrocarbon receptor signaling, xenobiotic metabolism signaling and G-protein coupled receptor signaling, further supporting the concept that waterpipe smoking has harmful effects on lung biology [24, 25]. There was also enrichment of pathways previously not associated with cigarette smoking and lung disease including cholecystokinin/gastrin-mediated signaling. The gastrointestinal peptides cholecystokinin (CCK) and gastrin are a structurally diverse group of secreted molecular messengers that regulate multiple normal and abnormal biological processes including development, inflammation, tissue regeneration, and neoplastic transformation [52]. Both CCK and gastrin exert their effects by binding to specific G-protein coupled receptors on the surface of a target cell and upon binding, trigger production of secondary messengers and subsequent Ca2+ release for activation of multiple kinase signal transduction pathways that relay the mitogenic signal to the nucleus [52, 53]. Important mediators that play a central role in relaying these activation signals related to cholecystokinin/gastrin-mediated signaling pathway include PLCB2 (phospholipase C, beta 2), PLCB3 (phospholipase C, beta 3), ITPR3 (inositol 1,4,5-trisphosphate receptor, type 3), RHOA (Ras homolog family member A), RHOF (Ras homolog family member F), SRC (src proto-oncogene), SOS2 (son of sevenless homolog 2), MAPK14 (mitogen-activated protein kinase 14) and the transcription factors JUN (jun proto-oncogene) and FOS (FBJ murine osteosarcoma viral oncogene homolog) [52], all of which display abnormal DNA methylation at the gene level in the SAE of waterpipe smokers *vs* nonsmokers. The role of cholecystokinin/gastrin-mediated signaling in human lung biology is unknown, however a recent study using a bleomycin-induced mouse model of pulmonary fibrosis demonstrated enrichment of this pathway in the target genes for altered miRNA expression in the fibrotic lung suggesting this pathway may play a role in the disease process [54]. Interestingly, some of the intracellular mediators for cholecystokinin/gastrin-mediated signaling also play a role in other signaling pathways including nicotine signaling [55]. Furthermore, multiple genes (e.g., FOS, JUN, PLCB2, PLCB3, SOS2 and SRC) displaying abnormal DNA methylation at the gene level in the SAE of waterpipe smokers *vs* nonsmokers were present in multiple pathways suggesting that waterpipe smoking may impact a wide variety of biological processes by disrupting a small number of key genes.

In addition to assessing the genome-wide DNA methylation changes associated with waterpipe smoking, we investigated whether these methylation changes were associated with alterations in the SAE transcriptome of the associated gene by correlating gene expression patterns from patient matched samples. Using this approach we identified 11.3% of the differentially methylated genes displayed a significant change in gene expression with pathway analysis of this gene set showing enrichment for the eIF2 signaling and regulation of eIF4 and p70S6K signaling pathways which play an important role in regulating mRNA translation and subsequent protein synthesis during homeostasis and in response to environmental stimuli [33–36]. Interestingly, four genes (EIF2S1, RPS5 and RPS15 and SOS2) displaying both DNA methylation and gene expression changes in the SAE of waterpipe smokers were present in both pathways suggesting possible functional redundancy in the biological roles of these pathways. Due to the critical role of proteins in regulating a large variety of biological processes, protein synthesis, folding and subsequent degradation (i.e. protein homeostasis or “proteostasis”) are fundamental to maintain optimal cellular function and tissue homeostasis during normal conditions and in response to environmental stress [56, 57]. This is of particular importance to the lung, which due to its anatomical structure is in direct contact with the outside world and continuously challenged by inhaled insults including cigarette smoke. One pathway critical for maintaining cellular homeostasis is termed the “integrated stress response” which is composed of four homologous stress-sensing kinases which are activated in response to cellular stress including protein folding efficiency in the endoplasmic reticulum (ER) and multiple environmental insults including viral infection and cigarette smoke [58–60]. In the presence of specific stress and/or insult, the integrated stress response is activated resulting in phosphorylation of the α subunit of eukaryotic translation initiation factor 2 (eIF2α) and subsequent inhibition of protein synthesis in the cell [33]. This global shutdown of protein synthesis serves a number of protective roles including relieving ER stress by reducing the rate of proteins entering the ER and allowing the unfolded protein response (UPR) to resolve the accumulation of misfolded proteins that compromise ER function [61, 62]. Phosphorylation of eIF2α promotes translation of a subset of mRNAs that help the cell adapt to these stresses including the transcription factor ATF4 (activating transcription factor 4) [33, 60–62]. A recent study in COPD identified a 98 gene airway gene expression signature which included many genes that were targets of the ATF4 transcription factor suggesting chronic activation of the integrated stress response abnormal proteostasis in COPD [63]. In addition to COPD, disruption of protein homeostasis has been associated with the development and progression of additional chronic lung diseases and genetic disorders including IPF, asthma, cystic fibrosis and α1-antitrypsin deficiency [59, 64–66]. The finding that light-use waterpipe smoking is associated with both methylation and transcriptomic changes in a number of genes linked to pathways that regulate protein translation and synthesis suggests waterpipe smoking impacts protein homeostasis of the SAE which may play a role in the pathogenesis of waterpipe-dependent lung disease.

### Impact and limitations of the study

We acknowledge the limitations of the study including the small sample population and lack of validation of the DNA methylation data by an independent methodology. Although the samples were all >98.6% pure epithelium, due to the small percentage (0.8–1.4%) of inflammatory cells in the SAE brushings we cannot rule out the possibility that some of the epigenetic and transcriptional changes we observe in response to waterpipe smoking are originating from non-epithelial cell populations. However, based on our knowledge, this is the first report regarding the impact of waterpipe smoking on epigenetics of the SAE, a cell population critical to the initiation and pathology of cigarette smoking induced lung disease. Due to the increasing use of waterpipe smoking among young adults and limited studies showing its biological and molecular impact on the lung, the results of this study will help in the design of larger epidemiologic and biologic studies on the harmful effects of waterpipe smoking.

In summary, the data demonstrates that light-use waterpipe smoking in young adults is associated with a broad range of genome-wide DNA methylation-related changes, with many of these methylation-related changes associated with changes in the SAE transcriptome. Relative to traditional cigarette smoking, waterpipe smoking impacts the SAE in both similar and unique ways at the DNA methylation level adding to the accumulating evidence that waterpipe smoking is harmful and detrimental to lung health.

## Supporting information

S1 Methods (DOC)

S1 Data File 727 differentially methylated probesets between waterpipe smokers and nonsmokers were identified representing 673 unique genes. (XLS)

S1 Fig Correlation analysis of the expression of 67 genes demonstrating differential DNA methylation and gene expression levels in the small airway epithelium of waterpipe smokers vs nonsmokers. (PDF)

S1 Table (DOCX)
