# Supplementary material for: Waterpipe smoking induces epigenetic changes in the small airway epithelium
Source: PLoS One. 2017 Mar 8;12(3):e0171112. doi: 10.1371/journal.pone.0171112 (PMC5342191; doi:10.1371/journal.pone.0171112)
Supplement: S1 Methods — (DOC) [file pone.0171112.s001.doc]

**Supplemental Methods**

**Inclusion and Exclusion Criteria**

**Nonsmokers**

**Inclusion criteria**

- Males and females, at least 18 years old
- Provide informed consent
- Normal physical examination
- Normal routine laboratory evaluation, including general hematologic studies, general serologic/immunologic studies, general biochemical analyses, and urine analysis
- HIV negative
- Good health without history of chronic lung disease, including asthma, and without recurrent or recent (within 3 months) acute pulmonary disease
- Normal PA and lateral chest X-ray
- Normal electrocardiogram (sinus bradycardia, premature atrial contractions are permissible)
- Not pregnant (females)
- No history of allergies to medications used in the bronchoscopy procedure
- Not taking any medications relevant to lung disease or having an effect on the airway epithelium or alveolar macrophages
- Willingness to participate in the study
- Self-reported never smokers, with smoking status validated by the absence of nicotine and cotinine in urine (nicotine <2 ng/ml and cotinine <5 ng/ml)

**Exclusion criteria**

- Unable to meet the inclusion criteria
- Current active infection or acute illness of any kind
- Evidence of malignancy within the past 5 years
- Alcohol or drug abuse within the past 6 months

**Waterpipe smokers**

**Inclusion criteria**

- Males and females, at least 18 years old
- Provide informed consent
- Normal physical examination
- Normal routine laboratory evaluation, including general hematologic studies, general serologic/immunologic studies, general biochemical analyses, and urine analysis
- HIV negative
- Good health without history of chronic lung disease, including asthma, and without recurrent or recent (within 3 months) acute pulmonary disease
- Normal PA and lateral chest X-ray
- Normal electrocardiogram (sinus bradycardia, premature atrial contractions are permissible)
- Not pregnant (females)
- No history of allergies to medications used in the bronchoscopy procedure
- Not taking any medications relevant to lung disease or having an effect on the airway epithelium or alveolar macrophages
- Willingness to participate in the study
- Self-reported waterpipe-only smokers

**Exclusion criteria**

- Unable to meet the inclusion criteria
- Current active infection or acute illness of any kind
- Evidence of malignancy within the past 5 years
- Alcohol or drug abuse within the past 6 months

**RNA Sequencing Gene Expression**

To validate the gene expression changes observed in the small airway epithelium (SAE) of waterpipe smokers *vs* nonsmokers, total RNA from the SAE of a subset of nonsmokers (n=3) and waterpipe smokers (n=3) were assessed using RNA sequencing on the Illumina HiSeq2500 following TruSeq v2 mRNA library prep. Paired-end reads were processed with STAR (version 2.3.1z13_r470) [1] for alignment to the GRCh37/hg19 human reference genome and RefSeq gene definitions (2014-06-02). Gene expression quantification was performed using Cufflinks (version 2.2) with conversion of aligned reads into fragments per kilobase of exon per million fragments sequenced (FPKM) using the same RefSeq gene definitions to correct for transcript length and coverage depth. The expression level of genes was characterized as FPKM ≥0.125 in every sample. The raw data are publically available at the Gene Expression Omnibus (GEO) site (http://www.ncbi.nlm.nih.gov/geo/), accession number GSE92662.

**References**

1. Dobin, A., Davis, C.A., Schlesinger, F., et al. (2013). STAR: ultrafast universal RNA-seq aligner. Bioinformatics. 29(1), 15-21
