# Supplementary material for: Waterpipe smoking induces epigenetic changes in the small airway epithelium
Source: PLoS One. 2017 Mar 8;12(3):e0171112. doi: 10.1371/journal.pone.0171112 (PMC5342191; doi:10.1371/journal.pone.0171112)
Supplement: S1 Fig — (PDF) [file pone.0171112.s003.pdf]

Supplemental Figure Legends

Supplemental Figure 1. Correlation analysis of the expression of 67 genes demonstrating differential DNA methylation and gene expression levels in the small airway epithelium of waterpipe smokers *v*s nonsmokers. Comparison of the waterpipe smokers *v*s nonsmokers $\log_2$ mean ratio in HG-U133 Plus 2.0 array gene expression (n=3 waterpipe smokers and n=3 nonsmokers) with RNA Sequencing gene expression from a subset of the same samples (n=3 waterpipe smokers and n=3 nonsmokers).

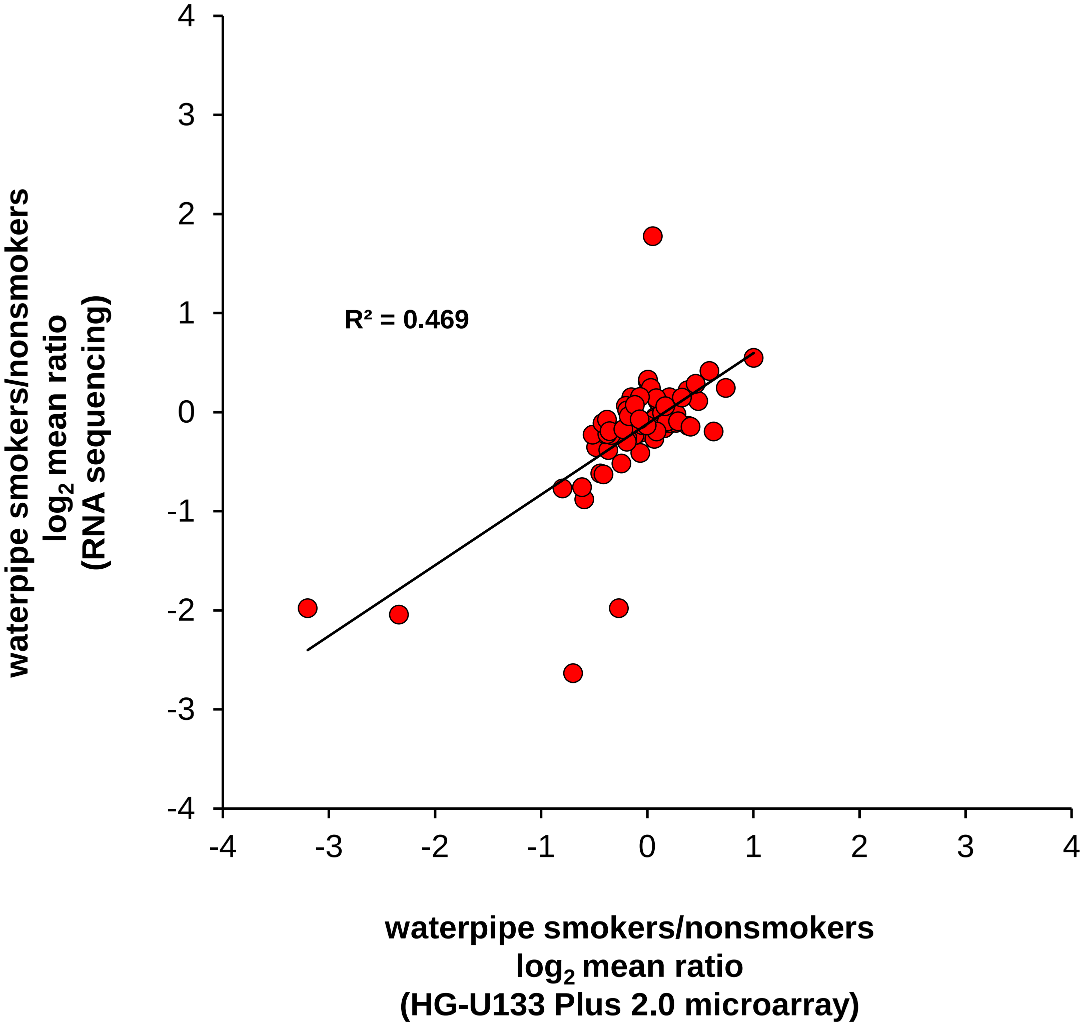

Supplemental Figure 1
