# Supplementary material for: Waterpipe smoking induces epigenetic changes in the small airway epithelium
Source: PLoS One. 2017 Mar 8;12(3):e0171112. doi: 10.1371/journal.pone.0171112 (PMC5342191; doi:10.1371/journal.pone.0171112)
Supplement: S1 Table — (DOCX) [file pone.0171112.s004.docx]

**S1 Table**

| **Sample number** | **Subject ID** | **Region** | **Phenotype** | **Cell differentials** | | | | | | **Total number of cells in brushings** | **Total number of epithelial cells in brushings** |
| --- | --- | --- | --- | --- | --- | --- | --- | --- | --- | --- | --- |
|  |  |  |  |  |  |  |  |  |  |  |  |
|  |  |  |  | **% inflammatory** | **% epithelial** | **% ciliated** | **% secretory** | **% basal** | **% intermediate** |  |  |
| 1 | DGM-10159_M12_sm | DLLL | nonsmoker | 4.3 | 95.7 | 61.5 | 8.8 | 12.5 | 14.2 | 5150000 | 4928550 |
| 2 | DGM-10565_M12_sm | DLLL | nonsmoker | 2.0 | 98.0 | 58.1 | 14.0 | 4.0 | 21.9 | 3450000 | 3381000 |
| 3 | DGM-10500_M6_sm | DLLL | nonsmoker | 0.3 | 99.7 | 52.5 | 7.8 | 20.9 | 18.5 | 8850000 | 8823450 |
| 4 | DGM-10503_M12_sm | DLLL | nonsmoker | 0.0 | 100.0 | 68.1 | 17.0 | 5.2 | 9.7 | 7750000 | 7750000 |
| 5 | DGM-10475_M3_sm | DLLL | nonsmoker | 1.4 | 98.6 | 64.2 | 4.6 | 15.0 | 16.5 | 5200000 | 5127200 |
| 6 | DGM-01774_M12_sm | DLLL | nonsmoker | 0.3 | 99.7 | 70.0 | 13.6 | 4.0 | 12.1 | 3800000 | 3788600 |
| 7 | DGM-00342_M0_sm | DLLL | nonsmoker | 1.6 | 98.4 | 74.7 | 2.8 | 1.6 | 19.3 | 5550000 | 5461200 |
| 8 | DGM-12697_M0_sm | DLLL | waterpipe smoker | 0.0 | 100.0 | 51.5 | 14.8 | 12.1 | 21.6 | 5350000 | 5350000 |
| 9 | DGM-12707_M0_sm | DLLL | waterpipe smoker | 0.0 | 100.0 | 64.8 | 21.4 | 0.9 | 13.0 | 5100000 | 5100000 |
| 10 | DGM-12715_M0_sm | DLLL | waterpipe smoker | 0.6 | 99.4 | 72.8 | 13.8 | 0.3 | 12.6 | 5400000 | 5367600 |
| 11 | DGM-12719_M0_sm | DLLL | waterpipe smoker | 3.3 | 96.7 | 74.5 | 4.8 | 2.1 | 15.2 | 3250000 | 3142750 |
| 12 | DGM-12750_M0_sm | DLLL | waterpipe smoker | 0.9 | 99.1 | 68.9 | 7.5 | 2.8 | 19.9 | 5450000 | 5400950 |
| 13 | DGM-12759_M0_sm | DRLL | waterpipe smoker | 0.6 | 99.4 | 70.7 | 11.0 | 2.4 | 15.2 | 4350000 | 4323900 |
| 14 | DGM-12775_M0_sm | DRLL | waterpipe smoker | 0.3 | 99.7 | 70.3 | 8.8 | 4.5 | 16.1 | 5850000 | 5832450 |
|  |  |  |  |  |  |  |  |  |  |  |  |
|  |  |  | **Average nonsmoker** | 1.4 | 98.6 | 64.2 | 9.8 | 9.0 | 16.0 | 5678571.4 | 5608571.4 |
|  |  |  | **Std Dev nonsmoker** | 1.5 | 1.5 | 7.5 | 5.2 | 7.2 | 4.3 | 1973545.3 | 1996304.8 |
|  |  |  | **Average waterpipe** | 0.8 | 99.2 | 67.6 | 11.7 | 3.6 | 16.2 | 4964285.7 | 4931092.9 |
|  |  |  | **Std Dev waterpipe** | 1.1 | 1.1 | 7.7 | 5.5 | 4.0 | 3.4 | 884455.7 | 912379.2 |
|  |  |  | **TTEST (2,3)** | 0.41 | 0.41 | 0.41 | 0.52 | 0.11 | 0.92 | 0.41 | 0.44 |
